# WHIRLY1 regulates aliphatic glucosinolate biosynthesis in early seedling development of Arabidopsis

**DOI:** 10.1111/tpj.17181

**Published:** 2024-12-03

**Authors:** Linh Thuy Nguyen, Pinelopi Moutesidi, Jörg Ziegler, Anike Glasneck, Solmaz Khosravi, Steffen Abel, Götz Hensel, Karin Krupinska, Klaus Humbeck

**Affiliations:** ^1^ Institute of Biology Martin‐Luther‐University Halle‐Wittenberg 06120 Halle (Saale) Germany; ^2^ Department of Molecular Signal Processing Leibniz Institute of Plant Biochemistry (IPB) 06120 Halle (Saale) Germany; ^3^ Program Center for Plant Metabolomics and Computational Biochemistry Leibniz Institute of Plant Biochemistry (IPB) 06120 Halle (Saale) Germany; ^4^ Institute of Botany Christian‐Albrechts‐University (CAU) 24098 Kiel Germany; ^5^ Department of Breeding Research Leibniz Institute of Plant Genetics and Crop Plant Research (IPK) Gatersleben 06466 Seeland Germany; ^6^ Centre for Plant Genome Engineering, Institute of Plant Biochemistry Heinrich‐Heine‐University Duesseldorf 40225 Duesseldorf Germany

**Keywords:** *WHIRLY1*, glucosinolate, seedling development, Arabidopsis

## Abstract

WHIRLY1 belongs to a family of plant‐specific transcription factors capable of binding DNA or RNA in all three plant cell compartments that contain genetic materials. In *Arabidopsis thaliana*, WHIRLY1 has been studied at the later stages of plant development, including flowering and leaf senescence, as well as in biotic and abiotic stress responses. In this study, *WHIRLY1* knockout mutants of *A. thaliana* were prepared by CRISPR/Cas9‐mediated genome editing to investigate the role of WHIRLY1 during early seedling development. The loss‐of‐function of *WHIRLY1* in 5‐day‐old seedlings did not cause differences in the phenotype and the photosynthetic performance of the emerging cotyledons compared with the wild type. Nevertheless, comparative RNA sequencing analysis revealed that the knockout of *WHIRLY1* affected the expression of a small but specific set of genes during this critical phase of development. About 110 genes were found to be significantly deregulated in the knockout mutant, wherein several genes involved in the early steps of aliphatic glucosinolate (GSL) biosynthesis were suppressed compared with wild‐type plants. The downregulation of these genes in *WHIRLY1* knockout lines led to decreased GSL contents in seedlings and in seeds. Since GSL catabolism mediated by myrosinases was not altered during seed‐to‐seedling transition, the results suggest that *AtWHIRLY1* plays a major role in modulation of aliphatic GSL biosynthesis during early seedling development. In addition, phylogenetic analysis revealed a coincidence between the evolution of methionine‐derived aliphatic GSLs and the addition of a new WHIRLY in core families of the plant order Brassicales.

## Significance Statement

*WHIRLY1* functions in several aspects of plant development and stress responses; however, little is known about its involvement in young seedling development. Our study shows that in this early stage, *WHIRLY1* regulates explicitly the expression of genes encoding enzymes involved in the early steps of aliphatic glucosinolate biosynthesis pathway, leading to a reduction in glucosinolate contents of the *WHIRLY1* knockout seeds and seedlings.

## INTRODUCTION

The WHIRLY protein family comprises few plant‐specific proteins sharing the eponymous single‐stranded (ss)‐DNA‐binding domain Whirly (Desveaux et al., 2000, 2002). The members of this family have the ability to bind preferentially to ssDNA but can also bind to dsDNA and RNA (Cappadocia et al., 2010; Desveaux et al., 2002). Each WHIRLY protein contains an N‐terminal organelle target peptide (OTP), which targets them into either mitochondria or chloroplasts. WHIRLY1 was reported to be targeted to chloroplasts (Desveaux et al., 2005; Isemer, Krause, et al., 2012) and also found in nucleus (Grabowski et al., 2008). By its dual localization in chloroplasts and nucleus, WHIRLY1 is an excellent candidate for the communication between organelles and the nucleus during plant development and stress responses (Krupinska et al., 2022; Taylor et al., 2022).

The first discovered WHIRLY protein was potato WHIRLY1, which was shown to form a whirligig‐like homo‐tetramer called PBF‐2. The binding of PBF‐2 to the elicitor response element (ERE; TGACAnnnnTGTCA) in the promoter of the potato pathogenesis‐related gene 10a (*StPR‐10a*) induced *StPR10a* expression in response to pathogen attack (Desveaux et al., 2000, 2002). Similarly, Arabidopsis WHIRLY1 can bind to the promoter of *PR1* and thereby influence plants' defense (Desveaux et al., 2004). In *Arabidopsis thaliana*, *WHIRLY1* TILLING mutants expressing mutated WHIRLY1 versions showed attenuated binding ability toward the *PR1* promoter, coinciding with reduced resistance against *Peronospora parasitica* (Desveaux et al., 2004). Moreover, *WHIRLY1* is involved in responses to biotic stresses in other species, such as maize (Kretschmer et al., 2017) and cassava (Liu et al., 2018).

WHIRLY1 can bind to an ERE‐like element overlapping with a W‐box in the promoter of *HvS40* in barley (Krupinska et al., 2014) and to the GNNNAAATT sequence plus an AT‐rich telomeric repeat‐like sequence in the *AtWRKY53* promoter in *A. thaliana* (Miao et al., 2013). Both genes function in leaf senescence (Humbeck et al., 1996; Miao et al., 2004) and are proposed to be negatively regulated by WHIRLY1. In addition to functions in pathogen response and leaf senescence, WHIRLY proteins are involved in abiotic stress responses, for example, drought, possibly via modulating histone markers in the promoter of stress‐related genes including *HvNCED1* (Janack et al., 2016; Manh et al., 2023). In fact, all three WHIRLIES in Arabidopsis were found to be associated to epigenetic reprogramming of meristem tissues of roots and shoots (McCoy et al., 2021). Furthermore, AtWHIRLY1 was shown to interact with HDA15 to modify histone markers of several flowering‐related genes (Huang et al., 2022).

The function of WHIRLY1 in early development was studied in monocots. *WHIRLY1* mutants of maize and rice exhibited severe disturbances in chloroplast development and consequently died at the three‐to‐four‐leaf stage (Prikryl et al., 2008; Qiu et al., 2022). In comparison, barley *WHIRLY1* knockout mutants survived despite severe disturbances in chloroplast development (Krupinska et al., 2024). In contrast, neither Arabidopsis *WHIRLY1* nor *WHIRLY2* T‐DNA insertion mutants show apparent phenotypes. It thus has been proposed that AtWHIRLY3 might replace AtWHIRLY1 or AtWHIRLY2 in the respective mutants (Krupinska et al., 2022). Analysis of the *why2* mutant line showed that germination and early seedling development were compromised (Golin et al., 2020; Negroni et al., 2024), whereas vegetative growth was unaffected. This result indicates that AtWHIRLY3 cannot replace all functions of AtWHIRLY2 during germination, which aligns with the low transcript abundance of *AtWHIRLY3* at this stage of development (Golin et al., 2020).

So far, research on the biological functions of Arabidopsis WHIRLY1 mainly concerned senescence and flowering. Still, little is known about the involvement of WHIRLY1 in early seedling development, which plays a vital role in the growth cycle. Therefore, this study aims to investigate whether AtWHIRLY1, similarly to AtWHIRLY2, plays a role in the early stage of plant life. Most previous studies on the function of AtWHIRLY1 used the T‐DNA insertion mutant line *why1‐1* (SALK_023713) featuring a T‐DNA integrated in a close proximity to the start codon (Huang et al., 2022; Isemer, Mulisch, et al., 2012; Lin et al., 2019), whereas, in the present work, new *WHIRLY1* knockout mutant lines prepared by CRISPR/Cas9‐mediated site‐directed mutagenesis were used to study functions of WHIRLY1 at the early stage of development. The loss‐of‐function of AtWHIRLY1 did not cause apparent changes regarding phenotype and photosynthetic performance of young seedlings. However, genome‐wide gene expression analysis revealed a significant impact of WHIRLY1 loss on the expression of a small set of nuclear genes. These genes encode enzymes involved in early steps of aliphatic glucosinolate (GSL) biosynthesis, changing aliphatic GSL abundances in the *WHIRLY1* knockout mutant compared with wild‐type seeds and seedlings. The involvement of At*WHIRLY1* in the regulation of aliphatic GSL‐related genes might be explained by the evolution of the WHIRLY family and GSL diversification in the order Brassicales.

## RESULTS

### CRISPR/Cas9‐mediated knockout mutants of *AtWHIRLY1*

**Figure 1:**
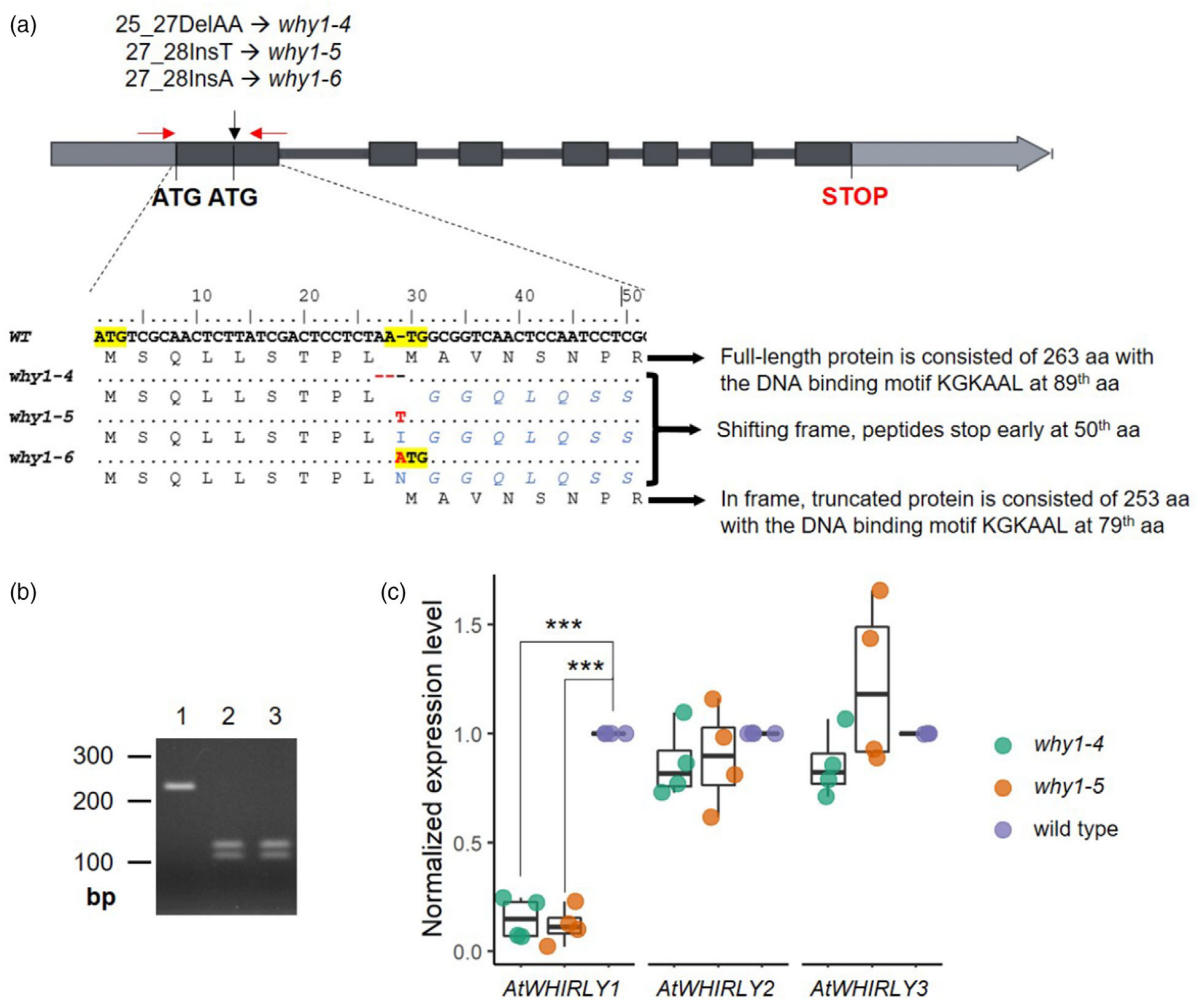
Genotyping of CRISPR/Cas9 mutants of *AtWHIRLY1*. (a) Scheme of three *WHIRLY1* knockout mutants used in the present work. The start codon (ATG) is highlighted and point mutations are depicted in red letters. Both *why1‐4* and *why1‐5* lines contain frameshift mutations, which change the protein sequence starting at 10th aa. The mutation in the w*hy1‐6* line might also cause frameshifting together with a novel ATG in frame within the wild‐type allele. Sizes of the expected mutant peptides encoded by these alleles are shown at the end of the alignment. (b) Two mutants, *why1‐4* and *why1‐5*, were verified using PCR‐coupled CAPS. The wild‐type allele gave a non‐cut fragment of 231 bp while CRISPR/Cas9‐mediated mutated alleles were digested into two products of 126 and 105 bp. The homologous mutants produce only two smaller bands. Lane 1: wild type, lane 2: *why1‐4*, lane 3: *why1‐5*. (c) Normalized expression level of three *WHIRLY* genes in two knockout mutants by qRT‐PCR compared with wild‐type plants. Boxplot central line shows median value, box limits indicate the 25th and 75th percentile, excluding outlier(s). Whiskers extend 1.5 times the interquartile range. Each dot represents the normalized mean value of each independent biological replicate, where a pool of about 30 seedlings was investigated. Gene expression level in the wild type was set as 1. Asterisks denote a statistically significant level of Student's *t*‐test between qRT‐PCR‐based normalized expression level of genes in the *WHIRLY1* knockout mutants and wild‐type plants (*n* = 4), ***P‐value <0.001.

So far, most studies on the AtWHIRLY1 function used the mutant line *why1‐1* having a T‐DNA insertion in the first exon (SALK_023713). This mutant had also been denominated KO‐1 (Yoo et al., 2007). However, in this mutant as well as two other T‐DNA insertion mutant lines, that is, *why1‐2* (SALK_147680; KO‐2 in Yoo et al., 2007) and *why1‐3* (SALK_03900), the mRNA levels of *AtWHIRLY1* were relatively high (Figure S1, Schaller, 2017). Noticeably, the *why1‐1* mutant line still has a second in‐frame ATG codon located downstream of the first ATG which might serve as an alternative start codon for the translation of a shorter protein lacking the first 10 amino acids of the plastid transit peptide. Hence, this truncated form of WHIRLY1 might be synthesized in *why1‐1* plants but mistargeted. To preclude this possibility, other *WHIRLY1* knockout mutants were prepared by CRISPR/Cas9‐mediated genome editing in the ecotype Col‐0 background. Three independent *Cas9*‐free homozygous mutant lines were generated and denotated as *why1‐4*, *why1‐5*, and *why1‐6*. *why1‐4 and why1‐5* mutant lines contain point mutations, 25_27delAA and 27_28insT, respectively, shifting the reading frame and altering the amino acid sequences of the residual proteins having 50 instead of 263 amino acids (Figure 1a). The mutation in the *why1‐*6 line, 27_28insA, resulted in a novel in‐frame ATG codon after the mutated site which might produce a truncated WHIRLY1 protein. This mutant thus was not investigated in this study to avoid the effects of such a truncated isoform. The two mutant lines, *why1‐4* and *why1‐5*, were verified using PCR‐coupled cleavage amplified polymorphic sequence analysis (Figure 1b), showing that these plants are homologous to the respective mutated alleles. Besides, the absence of a Cas9‐coding sequence was checked by PCR with specific primers (Table S4), showing that both lines are *Cas9*‐free. The knockout of At*WHIRLY1* by CRISPR/Cas9 resulted in a substantial reduction in the *WHIRLY1* transcript level compared with the wild type (Figure 1c), whereas expression levels of the two other *WHIRLY* genes were similar as in the wild type.

### Loss‐of‐function of *WHIRLY1* did not affect morphology and photosystem II efficiency

**Figure 2:**
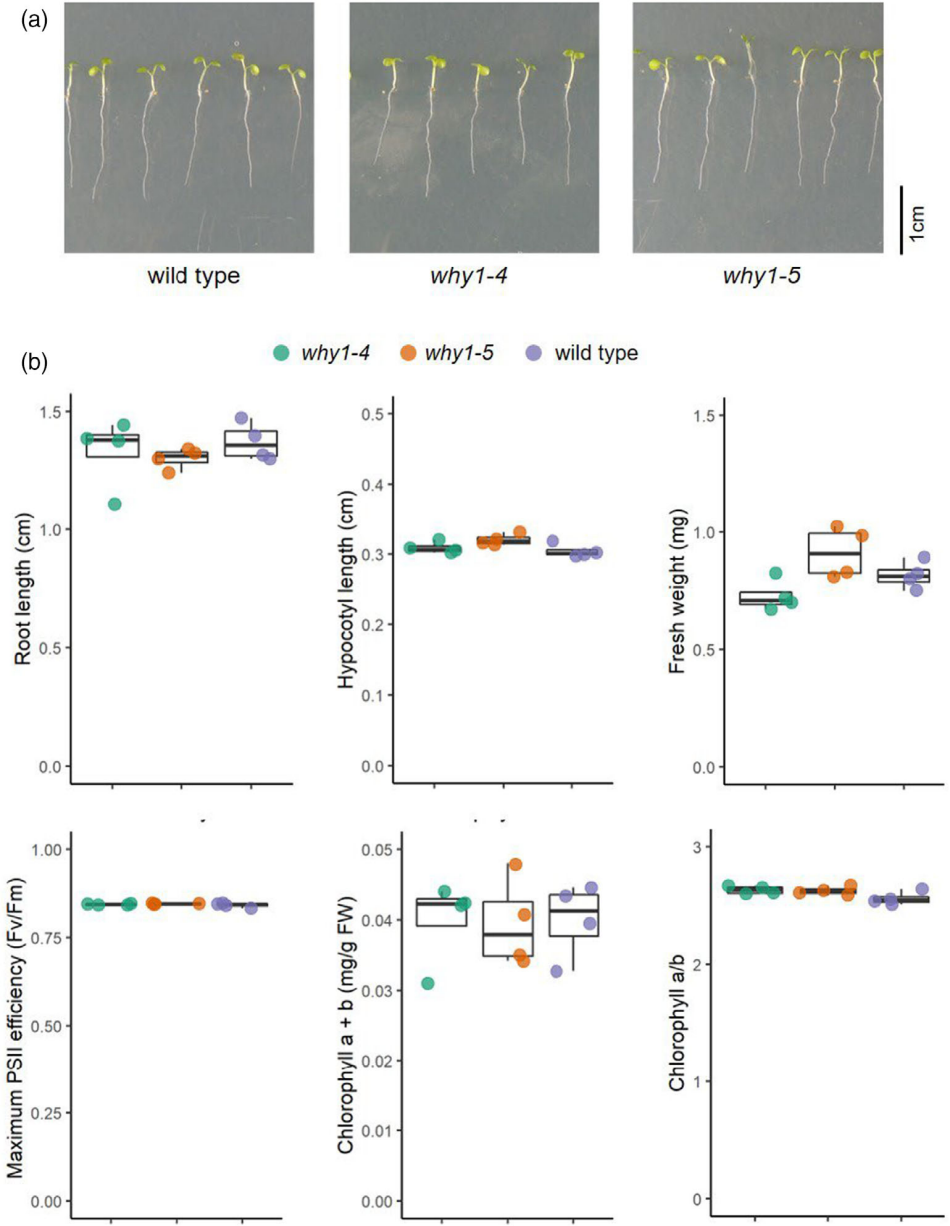
Phenotype of *WHIRLY1* knockout mutant seedlings. (a) Image of 5‐day‐old wild type and two CRISPR/Cas9 knockout mutant lines of *AtWHIRLY1*, *why1‐4* and *why1‐5*. (b) Root length, hypocotyl length, whole seedling fresh weight, maximum PSII efficiency (*F*_v_/*F*_m_), total chlorophyll a + b content, and chlorophyll a/b ratio of 5‐day‐old wild type and *why1* seedlings. Boxplot central line shows median value, box limits indicate the 25th and 75th percentile, excluding outlier(s). Whiskers extend 1.5 times the interquartile range. Each dot represents the mean value of each independent biological replicate by analyzing 20–30 seedlings. Asterisks indicate statistically significant level based on the Student's *t*‐test (*n* = 4), ***P‐value <0.001.

To investigate the impact of *WHIRLY1* knockout on seedling development, comparative phenotypic analysis of CRISPR/Cas9 knockout lines (*why1‐4* and *why1‐5*) and the wild‐type seedlings was performed. After 5 days grown on half‐strength MS medium, seedlings developed roots and green cotyledons (Figure 2a). Morphologically, there is no apparent difference between all lines regarding root length, hypocotyl length, and fresh weight (Figure 2b). Photosystem II efficiency indicated by the chlorophyll fluorescence parameter *F*_V_/*F*_M_ and chlorophyll content of cotyledons were similar between the wild type and mutant seedlings (Figure 2b). The results indicate that during early development under normal growth conditions, the loss‐of‐function of *WHIRLY1* has no evident influence on the vegetative growth and development of photosynthetic capacity.

### Loss‐of‐function of *WHIRLY1* specifically influenced the transcriptome of seedlings

To examine whether the absence of a functional version of *AtWHIRLY1* influences nuclear gene expression in 5‐day‐old seedlings, comparative transcriptome analysis by RNA sequencing (RNA‐seq) was performed between the knockout mutant line *why1‐5* and wild‐type plants. Total RNA isolated from about 30 seedlings grown on the same agar plate was treated as one biological replicate. The approach consists of three independent biological replicates per sample. A summary of read numbers and mapping rates is presented in Table S1.

**Figure 3:**
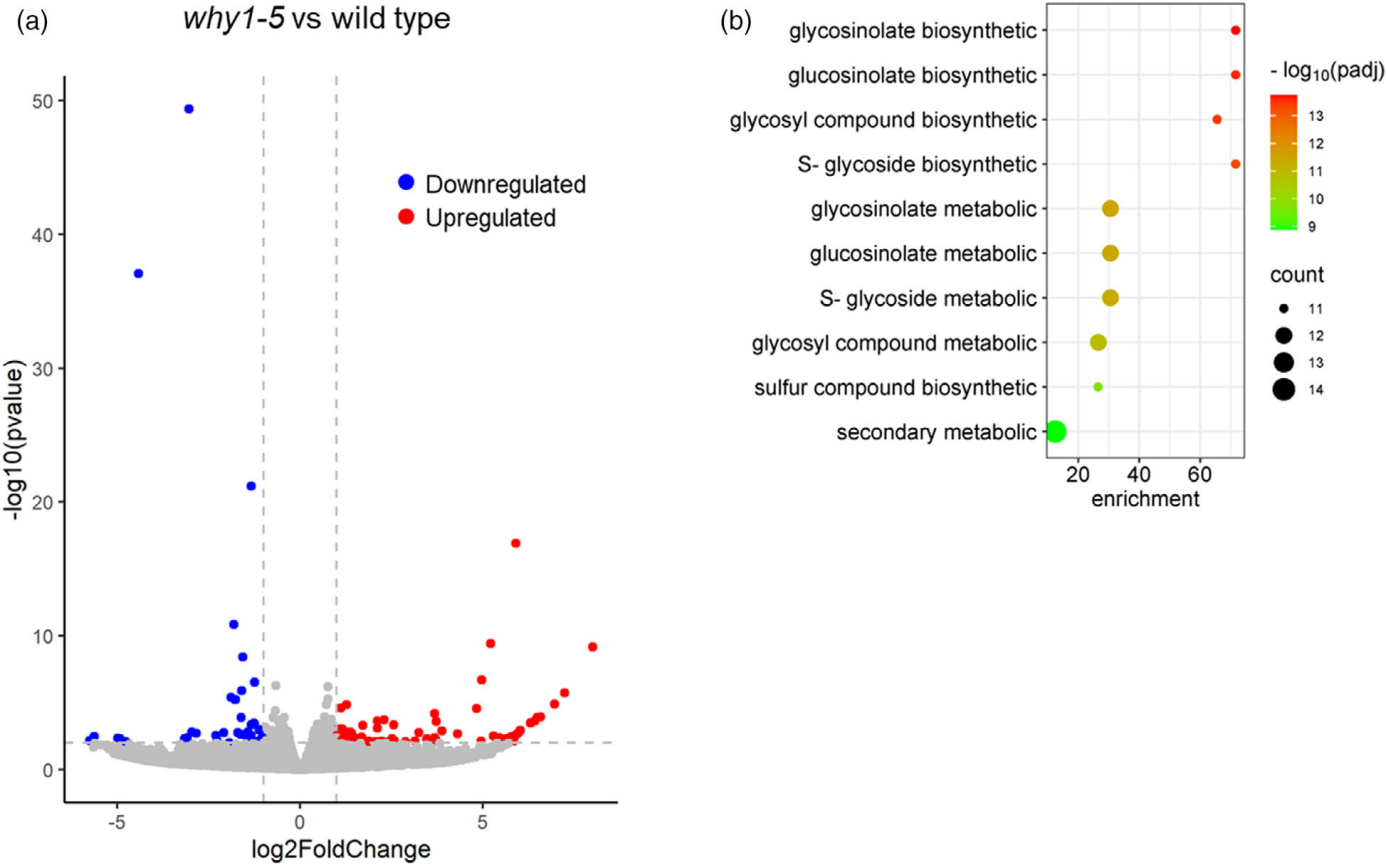
Comparative transcriptomic analysis between *why1‐5* mutant and wild‐seedlings. (a) Differentially expressed genes (DEGs) with |log_2_FC| > 1 and *P*‐value <0.01 in the *why1‐5* mutant compared with the wild‐type seedlings are shown by a Volcano plot. (b) Gene Ontology (GO) enrichment analysis of downregulated DEGs. The top 10 GO biological process terms according to *P*‐adjusted value are shown.

In each line, nearly 15 thousand genes were expressed as their expression levels were above the threshold, that is, fpkm (fragment per kilobase of transcript per million mapped reads) value >1. The differences in gene expression between *why1‐5* and wild‐type seedlings were identified using DESeq2 program. Considering that mutant seedlings did not show apparent changes in morphology and photosystem II efficiency compared with the wild‐type plants, differentially expressed genes (DEGs) were evaluated using a mild stringent cut‐off of |log_2_fold‐change(FC)| > 1 and *P*‐value <0.01. As a result, 73 upregulated DEGs and 42 downregulated DEGs were identified in the *why1‐5* mutant compared with wild‐type seedlings, indicating that WHIRLY1 can exert both positive and negative effects on target gene transcript abundances. The low number of DEGs (Figure 3a) also implies that the loss‐of‐function of *WHIRLY1* has no profound influence on the transcriptome during this early growth stage, but rather has a specific impact on a small gene set. Gene Ontology (GO) enrichment analysis failed to identify significant GO terms for over‐accumulated transcripts in *why1‐5* compared with wild type. However, by this approach, a small set of 42 significantly downregulated DEGs upon *WHIRLY1* knockout was enriched with genes related to the metabolism of sulfur‐containing compounds. In particular, the GSL biosynthesis ontology term showed a rather high enrichment fold (Figure 3b). Besides GO enrichment analysis, a manual search for all up‐ and downregulated DEGs in public databases (The Arabidopsis Information Resource (TAIR), www.arabidopsis.org and UniProt, https://www.uniprot.org/) revealed that multiple DEGs upon *WHIRLY1* knockout are associated to various other processes, including development and stress responses (Table S2).

### Downregulation of genes encoding enzymes of the aliphatic glucosinolate pathway

**Figure 4:**
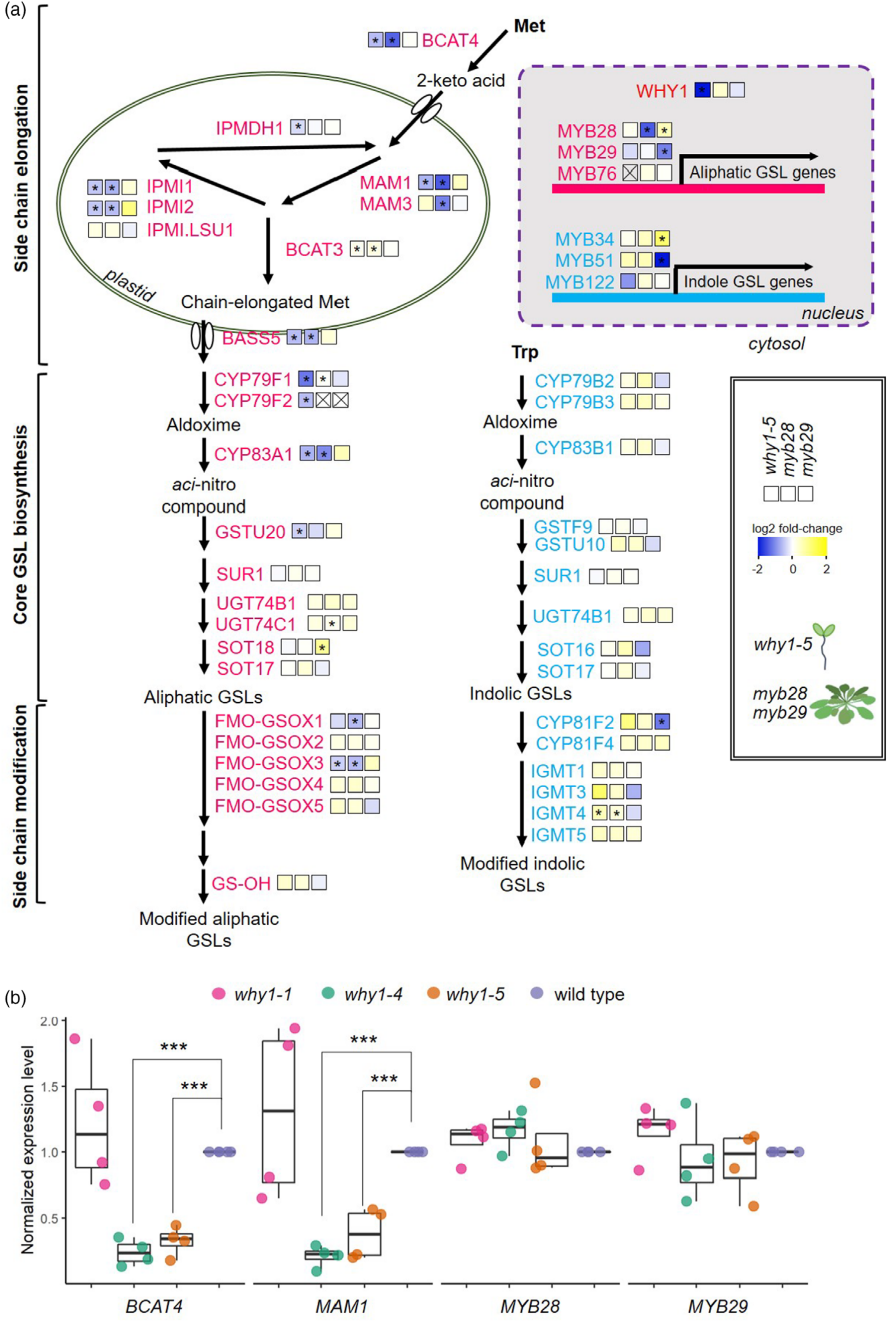
Loss‐of‐function of *WHIRLY1* affects glucosinolate (GSL)‐related gene expression. (a) Heatmap showing changes in transcript levels of GSL‐related genes due to the knockout of *WHIRLY1* and due to knockout of *MYB28* and *MYB29* (Sønderby et al., 2010). The log_2_FC is indicated by color bar, and asterisks (*) show statistically significant differences as a result of the RNA‐seq or microarray. The scheme of two major GSL biosynthesis pathways, aliphatic GSL and indole GSL, and also for gene names have been adapted from Augustine and Bisht (2017). See there also for gene abbreviations. (b) Normalized expression levels of selected GSL‐related genes measured by qRT‐PCR. Boxplot central line shows median value, box limits indicate the 25th and 75th percentile, excluding outlier(s). Whiskers extend 1.5 times the interquartile range. Each dot represents the normalized mean value of each independent biological replicate, where a pool of about 30 seedlings was investigated. Gene expression level in wild type was set as 1. Asterisks indicate statistically significant level of Student's *t*‐test between *WHIRLY1* knockout mutants and wild‐type plants (*n* = 4); *P‐value <0.05; **P‐value <0.01; ***P‐value <0.001.

A striking result of the comparative transcriptomic analysis was that 10 downregulated DEGs are involved in GSL biosynthesis (Figure 3b). GSLs are secondary metabolites composed of a thioglucose, a sulfate group, and a variable side chain derived from amino acids (Mérillon & Ramawat, 2017). With regard to the precursor amino acid, GSLs are categorized into three groups: aliphatic glucosinolate (aGSL), indole glucosinolates (iGSL), and benzenic glucosinolate (bGSL). As illustrated in Figure 4(a), aGSL biosynthesis involves three main stages: side‐chain elongation occurring in the plastid, core‐structure formation, and side‐chain modification, both of which take place in the cytosol.

According to the transcriptomic data, loss‐of‐function of WHIRLY1 specifically affected the transcript abundances of several genes encoding enzymes in the early steps of methionine (Met)‐derived aGSL biosynthesis, such as *BRANCHED‐CHAIN AMINOTRANSFERASE 4* (*BCAT4*), *METHYLTHIOALKYLMALATE SYNTHASE 1* (*MAM1*), *ISOPROPYLMALATE ISOMERASE 1* (*IPMI1*), and *BILE ACID:SODIUM SYMPORTER 5* (*BASS5*), of which most are plastid‐localized (Figure 4a). Additionally, genes encoding enzymes in later stages of GSL biosynthesis were also suppressed in the *why1‐5* mutant line compared with wild type, such as *CYTOCHROME P450 79F1 (CYP79F1)*, *CYP79F2*, and *CYP83A1* (Figure 4a). The downregulation of the aforementioned genes in *WHIRLY1* knockout seedlings was validated by qRT‐PCR, showing a high correlation between RNA‐seq and qRT‐PCR data (Figure S2). The expression levels of numerous genes related to the biosynthesis of aGSL in the *why1‐5* seedlings were substantially decreased and were about a third of the wild‐type levels (Figure S2). In contrast, the expression of most genes involved in iGSL biosynthesis was not affected in the knockout line *why1‐5* compared with wild type (Figure S2). Only *SUPERROOT1* (*SUR1*), a gene shared between aGSL and iGSL pathways, showed slightly reduced transcript levels in the *why1‐*5 mutant compared with wild‐type seedlings. The downregulation of selected aGSL biosynthesis‐related genes was also confirmed in seedlings of the other CRISPR/Cas9‐mediated knockout line *why1‐4* (Figure 4b). Interestingly, in contrast to the CRISPR/Cas9 knockout mutants, the T‐DNA insertion mutant *why1‐1* had a similar expression level of GSL‐related genes as the wild‐type plants, indicating that different mutated versions of *WHIRLY1* can result in distinctive impacts on nuclear gene expression (Figure 4b).

Biosynthesis of aGSL is known to be directly controlled by MYELOBLASTOSIS transcription factors (MYBs), including MYB28, MYB29, and MYB76, while iGSL biosynthesis is governed by MYB34, MYB51, and MYB122 (Mérillon & Ramawat, 2017). Analyses of the expression of the corresponding genes in the *why1‐5* mutant and wild‐type seedlings revealed no differences between both lines (Figure 4b; Figure S2). The findings indicate that WHIRLY1 does not regulate aGSL biosynthesis genes by indirectly modulating transcription of these upstream regulators, that is, *MYB28* and *MYB29*, but likely via another mechanism.

Transcription alteration of GSL‐related genes in seedlings upon *WHIRLY1* knockout was then compared with the expression changes in respective genes in knockout mature plants of *MYB28* and *MYB29* using the available RNA‐array data (Sønderby et al., 2010). Interestingly, similar changes in the expression levels of GSL biosynthesis genes were observed in the *myb28* and *why1‐5* line compared with wild‐type plants (Figure 4a). In contrast to these two mutants, the *myb29* showed a different pattern of the expression of GSL‐related genes. In contrast to the knockout of *MYB29*, *MYB28* loss‐of‐function led to repression of same genes involved in side‐chain elongation steps as in the knockout *why1‐5* mutant. For instance, *BCAT4*, which encodes the key enzyme initiating the side‐chain elongation process, was significantly suppressed in the knockout lines *why1‐5* and *myb28* compared with wild‐type plants, while *BCAT4* transcript levels did not alter upon *MYB29* knockout. On the contrary, genes encoding enzymes in the iGSL pathway were slightly but not significantly induced in both *why1‐5* and *myb28* lines compared with the wild type. In contrast, some genes, such as *MYB34* and *CYP81F2*, were considerably deregulated in the *myb29* mutant. Interestingly, the effect of the loss‐of‐function of WHIRLY1 on aGSL‐related gene expression was only observed in the seedling stage but not in the rosette of mature plants (Figure S3).

### Loss‐of‐function of WHIRLY1 altered GSL contents in the mutant

**Figure 5:**
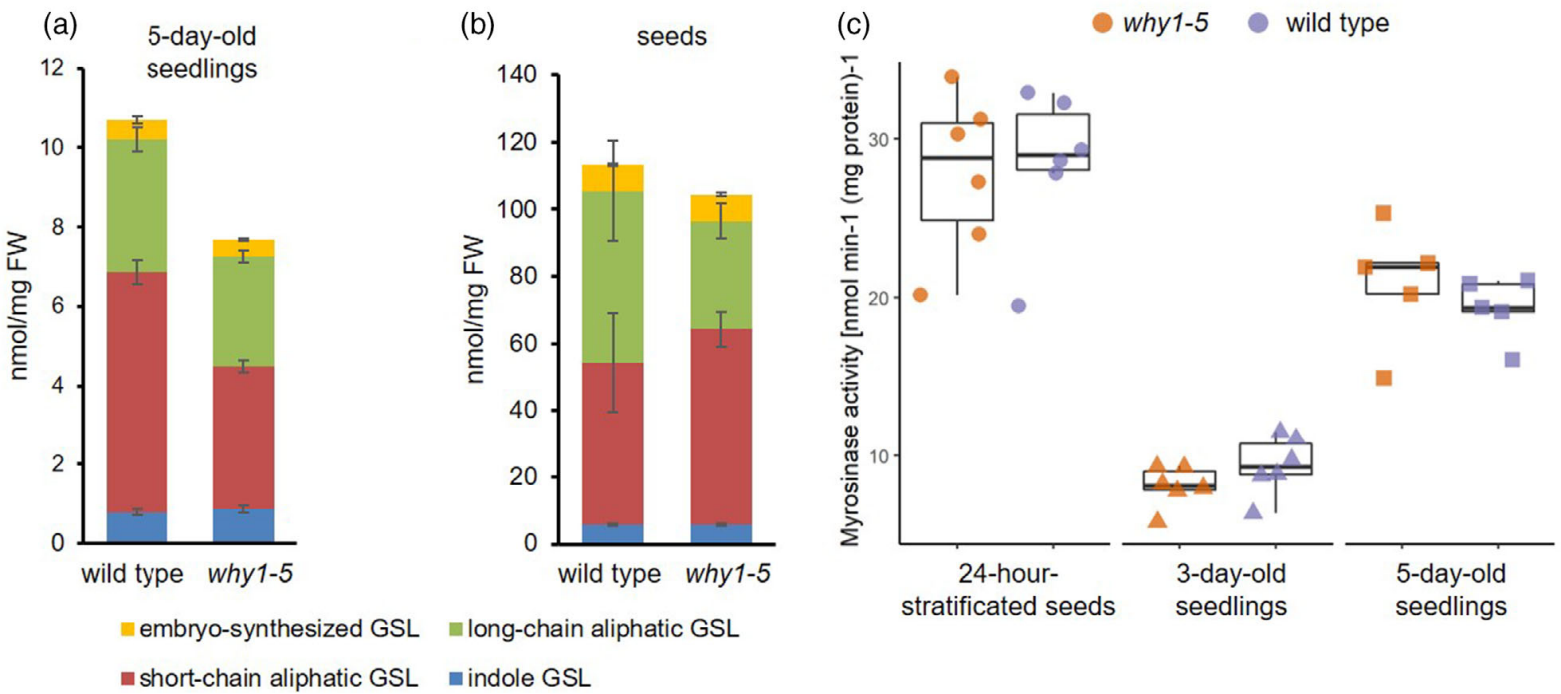
Glucosinolate contents and myrosinase activities in the *WHIRLY1* knockout and wild‐type plants. (a, b) Glucosinolate contents (nmol/mg FW) of indole glucosinolate (GSL), short‐chain aliphatic GSL, long‐chain aliphatic GSL, and embryo‐synthesized GSL (3‐BZOP) measured by HPLC‐MS/MS. Bar charts show the average and error bars show standard deviation of (a) three independent biological replicates, each containing four samples as pool of 5‐day‐old seedlings and (b) six independent samples, each containing around 5 mg seeds. (c) Myrosinase activity [nmol min^−1^ (mg protein)^−1^] of 24‐h‐stratified seeds, 3‐day‐old, and 5‐day‐old seedlings. Boxplot central line shows median value, box limits indicate the 25th and 75th percentile, excluding outlier(s). Whiskers extend 1.5 times the interquartile range. Each dot represents the mean value of each independent biological replicate, whereby around 100 mg of fresh material as a pool of seeds/seedlings was investigated (*n* = 5–6).

To investigate whether the specific suppression of genes encoding enzymes in the early steps of aGSL biosynthesis influences aGSL contents, 5‐day‐old *why1‐5* and wild‐type seedlings were collected to quantify GSLs by LC–MS/MS. The amount of each GSL in each sample was calibrated using sinigrin, a compound not naturally found in *A. thaliana*, and then normalized to sample fresh weight. GSL profiles of the knockout *why1‐5* and wild‐type seedlings were in line with the transcriptomics data (Figure 5a; Table S3). In 5‐day‐old *why1‐5* seedlings, the total GSL content was significantly lower than that in wild type, resulting from significantly reduced abundances of individual short‐chain aGSL compounds (Figure 5a; Table S3). For example, the wild‐type seedlings contained almost twice as much 4‐methylthio‐3‐butenyl (4MTB) as the *why1‐5* mutant seedlings (2.92 nmol mg^−1^ FW versus 5.02 nmol mg^−1^ FW; Table S3). For comparison, long‐chain aGSLs were only reduced mildly due to the knockout of *WHIRLY1* (Figure 5a). Furthermore, indole GSL and embryo‐synthesized GSL concentrations were similar between these two lines.

It has been reported that in young seedlings, the majority of GSLs are from those synthesized and accumulated during embryo development while a minority is newly synthesized after germination (Jeschke et al., 2019; Meier et al., 2019). To clarify whether absence of the functional WHIRLY1 already disturbs GSL composition in seeds, GSL contents of dry seeds were quantified and compared between the *why1‐5* mutant and wild‐type line (Figure 5b; Table S4). The data show that seeds have a much higher total GSL level than seedlings (Figure 5b), which is in line with other reports (Meier et al., 2019; Mérillon & Ramawat, 2017; Wittstock & Burow, 2010). As in seedlings, knockout of *WHIRLY1* specifically affected aliphatic GSL content but did not influence indole as well as embryo‐synthesized GSLs, leading to an overall lower total GSL amount in the *why1‐5* compared with wild‐type seeds. This result suggests that *WHIRLY1* might also act as a positive regulator of aGSL biosynthesis‐related genes in the embryo and seed development. Interestingly, the quantity of short‐chain aGSL was significantly higher in the *why1‐5* than in the wild‐type seeds, while long‐chain aGSL contents showed an opposite tendency (Figure 5b; Table S4). Perhaps, the side‐chain elongation of methionine (first stage of aGSL biosynthesis) is impaired in the mutant, resulting in a higher portion of short‐chain aGSLs than long‐chain aGSLs.

### Myrosinase activity during seed‐to‐seedling transition is not affected by the loss‐of‐function of *WHIRLY1*

During the seed‐to‐seedling transition, GSLs in seeds are turned over to provide building blocks for the development of seedlings. GSLs are enzymatically hydrolyzed by the action of either classical myrosinases (TGG1‐6) or atypical myrosinases called beta‐glucosidase enzymes (BGLUs), both exhibiting myrosinase activities (Barth & Jander, 2006; Meier et al., 2019; Sugiyama et al., 2021; Sugiyama & Hirai, 2019). The discrepancy in total GSL content between *why1‐5* and the wild type during this transition raised the question whether the lower aGSL concentration in mutant seedlings is caused by altered biosynthesis and/or by differences in catabolism by myrosinases. To answer this question, overall myrosinase activities in 24‐h‐stratified seeds and in seedlings 3 and 5 days after germination were measured as the rate of sinigrin degradation (Figure 5c). Protein extracts from imbibed seeds showed the highest activity of myrosinases among investigated samples. In comparison, seedlings exhibited a lower myrosinase activity than seeds, whereby the older seedlings seemed to have more myrosinase activity than the younger ones. Interestingly, there was no difference in myrosinase activity between the *why1‐5* mutant and the wild‐type samples. The results indicate that the difference in aGSL content between *why1‐5* and wild‐type seedlings is not caused by altered GSL degradation driven by myrosinases. This does not exclude that other myrosinase‐independent breakdown mechanisms of GSLs are affected.

### The evolution of WHIRLY in the plant order Brassicales coincides with glucosinolate diversification

The number of *WHIRLY* genes and proteins has been investigated restricted to a limited number of species (Desveaux et al., 2002; Muti et al., 2024; Oetke et al., 2022; Qiu et al., 2022). So far, a comprehensive phylogenetic analysis of the WHIRLY family has not been performed. Thanks to the increasing number of plant genomes sequenced in recent years, it is feasible to obtain WHIRLY sequences and to analyze the evolution of the WHIRLY family. Thousand sequences sharing similarity in either amino acid sequence or higher protein structure were identified using the Whirly domain sequence of AtWHIRLY1 to perform BLAST searches against NCBI and Pfam databases. After removing duplications, isoforms, and poor‐quality sequences, 772 sequences of 371 species were retained. It is worth noting that with this procedure, some highly homologous proteins derived from respective loci as resulted of whole‐genome duplication might be excluded. Sequences were then aligned and used to build a phylogeny tree of the WHIRLY family (Figure S4).

The evolutionary tree revealed that WHIRLY proteins can be grouped into two distinctive groups, WHIRLY1‐like and WHIRLY2‐like (Figure S4). Most angiosperm plant species contain only these two proteins, such as in barley (Grabowski et al., 2008), maize (Prikryl et al., 2008), and tomato (Akbudak & Filiz, 2019). Interestingly, the phylogenetic tree of WHIRLY family revealed that several plant lineages have an additional ortholog of either WHIRLY1 or WHIRLY2. Besides the Brassicaceae, Cucurbitaceae and Salicaceae have two distinctive proteins in the WHIRLY1‐like group supported by high bootstrap values (Figure S4). In the case of the Fabaceae family, two clades represent two separated WHIRLY1‐like proteins (denoted WHIRLY1 and WHIRLY3 in Figure S4), although the bootstrap value is relatively low, likely due to the high variance in protein sequences within this family. Moreover, it is worth noting that in complex genomes, such as in polyploid, the number of *WHIRLY* loci is higher but their products are only grouped into two distinctive clades, each representing either WHIRLY1‐like or WHIRLY2‐like proteins, respectively (Hu & Shu, 2021). The appearance of additional WHIRLY proteins in plant groups, that are quite different in physiological characteristics and ecological adaptations, can be explained by evolutionary processes, leading to new proteins which can gain new function(s).

**Figure 6:**
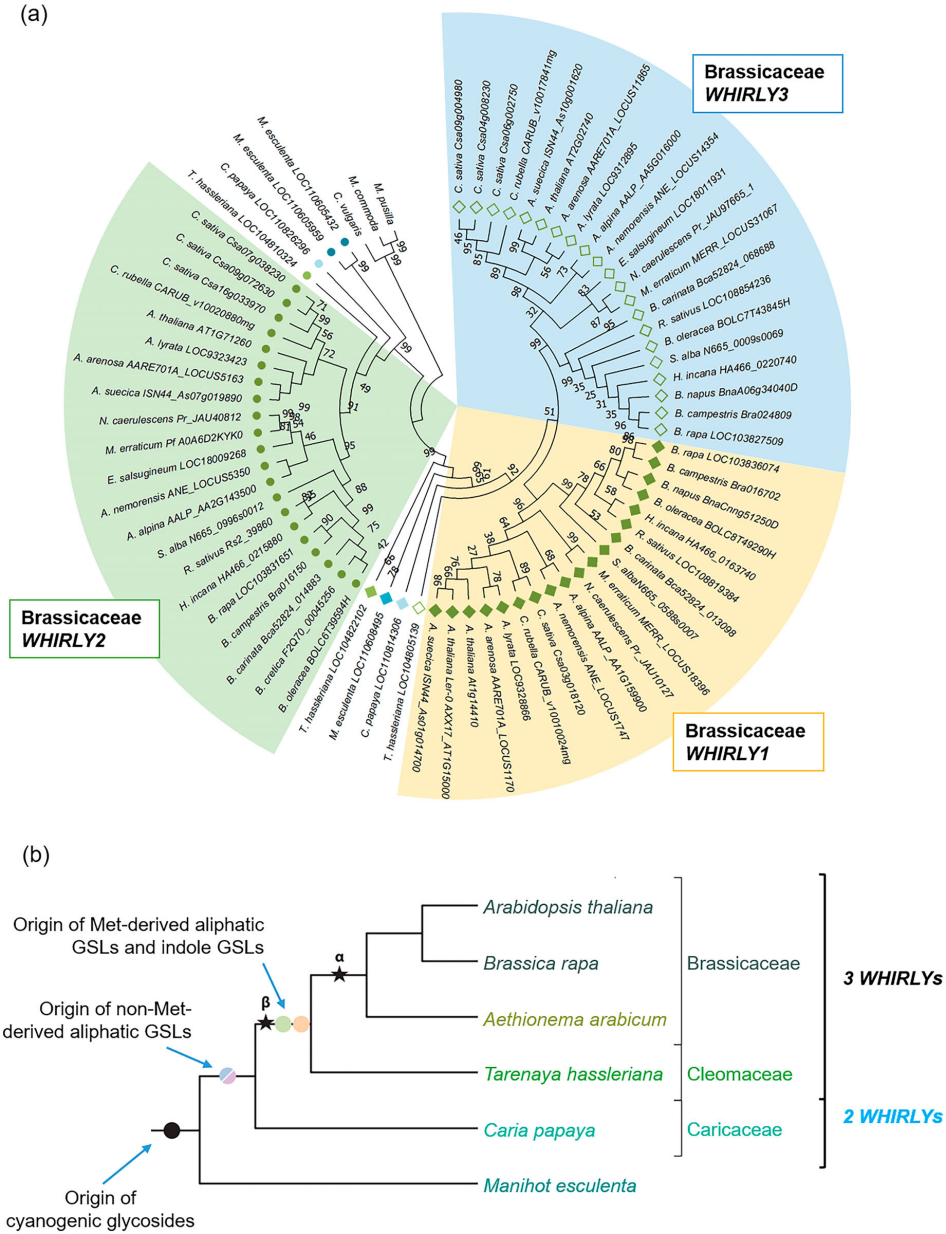
Evolution of WHIRLY and glucosinolate (GSL) diversification in the order Brassicales. (a) The evolutionary history of the WHIRLY family in the order Brassicales was constructed using the neighbor‐joining method. The rooted bootstrap consensus tree inferred from 1000 replicates, in which the bootstrap values are shown next to the branches. The analysis involved 72 protein sequences, including WHIRLY proteins in Brassicales species and *Morchella esculenta*, and putative WHIRLY‐like proteins of algae as outgroup. Name of species and gene/locus are shown at the tip of each branch. Closed circles represent WHIRLY2, while closed and opened diamonds illustrate WHIRLY1 and WHIRLY3, respectively. (b) The dendogram depicted major evolutionary events in GSL diversity uniquely in Brassicales. Many plant groups can produce cyanogenic glycosides as defense compounds but are uncapable of metabolizing GSLs, such as *M. esculenta*. Non‐Met‐derived aliphatic GSL pathway occurred in Brassicales plants as they separated from other dicots. Other GSL groups and their biosynthesis appeared after two whole‐genome duplication events (WGD). Thus, only core plant families in Brassicales can produce Met‐derived aliphatic GSLs. Circles represent the origin of novel GSL groups and stars illustrate WGD events. Number of WHIRLY proteins in these families (according to a) are shown on the right. The scheme was adapted from Barco and Clay (2019).

Further investigating the order Brassicales showed that three WHIRLY proteins are found in numerous genera, including the genera *Arabidopsis* and *Brassica* of the family Brassicaceae, and *Tarenaya* of the family Cleomaceae (Figure 6a). Interestingly, in *Carica papaya*, a species of the family Caricaceae, only two WHIRLIES are identified (Figure 6a). The additional third WHIRLY in core families of Brassicales can be explained by the evolutionary history of this plant order. Brassicales evolved thanks to two whole‐genome duplication events (WGD) designated the At‐α and the At‐β (Figure 6b) (Edger et al., 2018). It is likely that *Carica* plants do not have an additional *WHIRLY* locus because of its emergence happened before these WGD events. In contrast, *T. hassleriana* appearing right after the WGD event At‐β has already two WHIRLY1‐like proteins, which are different from others and from Brassicaceae WHIRLY1/3 proteins (Figure 6a). Interestingly, the recent WGD event At‐α did not further increase the number of *WHIRLY* loci among Brassicaceae species.

The diversity of GSLs regarding their chemical properties is also a result of WGD events and the neofunctionalization of genes (Figure 6b). Gene duplication at the At‐β was the origin of novel iGSL and Met‐derived aGSL pathways (Barco & Clay, 2019). Whereas the more recent WGD event At‐α gave rise to many side‐chain modification genes for both iGSLs and Met‐derived aGSLs. Therefore, Met‐derived aGSLs are only present in core families of the order Brassicales, that is, those derived after the event At‐β, such as Brassicaceae, Cleomaceae, etc. (Mérillon & Ramawat, 2017). The family Caricaceae, which has two WHIRLIES, contains only non‐Met‐derived GSL (Mérillon & Ramawat, 2017). It is tempting to speculate that the coincidence between the third WHIRLY1‐like protein and the aliphatic GSL biosynthesis pathway provides an evolutionary explanation for the function of *WHIRLY1* in the regulation of aGSL biosynthesis.

## DISCUSSION

### Knockout of *WHIRLY1* has no impact on seedling development but specifically alters gene expression

WHIRLY1 has been described as a multifaceted transcription regulator that modulates plant development and stress responses (Krupinska et al., 2022; Taylor et al., 2022). In this study, novel *AtWHIRLY1* mutants were generated using CRISPR/Cas9‐mediated site‐directed mutagenesis. These knockout lines have point mutations which cause a frameshift, changing the protein sequence starting from the eleventh amino acid, thereby resulting in relatively short and likely unfunctional peptides (Figure 1a). Despite the true knockout of *AtWHIRLY1*, these mutants did not show an obvious phenotypic alteration during seedling development, suggesting that the presence of WHIRLY1 is seemingly unnecessary for seedling growth under standard growth conditions. The lack of an apparent phenotype can be explained by an unknown mechanism compensating *WHIRLY1*‐knockout effects on chloroplast performance and development of seedlings. This complementation in Arabidopsis *WHIRLY1* knockout mutants could be done preferably by AtWHIRLY3, which was shown to be dually targeted into both chloroplast and mitochondria (Golin et al., 2020). It has been reported that the expression level of *AtWHIRLY3* was enhanced in shoots of *why2* knockout mutant at the two‐ to four‐true‐leaf stage, suggesting a compensation of WHIRLY2 deficiency by enhanced abundance of WHIRLY3 (Golin et al., 2020). In the same manner, it is likely that AtWHIRLY3 might also replace AtWHIRLY1 in seedling development in the *WHIRLY1* knockout mutants. Indeed, AtWHIRLY1 and AtWHIRLY3 share 77% similarity in protein sequences and might have similar functions under certain conditions (Desveaux et al., 2004). For comparison, monocot plants with WHIRLY1 deficiency show obvious phenotypes likely due to lack of a third WHIRLY protein. In barley *WHIRLY1* RNAi‐mediated knockdown and CRISPR/Cas9‐mediated knockout mutants, chloroplast development is retarded coinciding with suppressed development of photosynthetic function and delayed formation of green tissue, leading to prolonged overall development (Krupinska et al., 2019, 2024; Saeid Nia et al., 2023). *WHIRLY1* mutants of maize and rice are seedling‐lethal mutants that can form only a few albino leaves (Prikryl et al., 2008; Qiu et al., 2022).

Although the CRISPR/Cas9‐mediated *WHIRLY1* knockout mutants lack an apparent phenotype, RNA‐seq analysis of the *why1‐5* mutant seedlings revealed distinct alterations in nuclear gene expression, indicating that WHIRLY1 in this stage is involved specifically in the regulation of a small set of nuclear genes. These target genes of WHIRLY1 have functions in various developmental processes and stress responses (Table S2). Among the genes differentially expressed in the *why1‐5* mutant compared with the wild type, genes encoding enzymes of aliphatic GSL biosynthesis were significantly suppressed (Figure 4). Interestingly, the previously described target genes of WHIRLY1 in mature plants, including *AtPR1* (Desveaux et al., 2004) and *AtWRKY53* (Miao et al., 2013), which were discovered using other mutants than the CRISPR/Cas lines, were not differently expressed in seedlings of the *why1‐*5 mutant. This could be interpreted in two ways; one would be that WHIRLY1 may perform various functions in many developmental stages via mediating the expression of different target genes. Another explanation could be that differently mutated WHIRLY1 proteins in the respective mutants affect gene expression in different ways.

### Participation of WHIRLY1 in the regulation of glucosinolate homeostasis during seedling development

Comparative transcriptomic analysis of 5‐day‐old seedlings revealed that *WHIRLY1* knockout caused reduced transcript accumulation of several genes involved in aGSL biosynthesis (Figure 4), indicating that WHIRLY1 acts as a positive regulator of these genes. Consequently, aGSL contents were lower in the *why1‐5* compared with wild‐type seedlings (Figure 5a). Most GSLs in seedlings are already synthesized and accumulated in the embryos and serve as a nutrient reservoir for growing seedlings (Wittstock & Burow, 2010). During germination, GSLs are degraded, and sulfur and other nutrients are remobilized for the growing seedlings (Meier et al., 2019; Sugiyama et al., 2021). This leads to a substantially reduced GSL concentration during the seed‐seedling transition. As in seedlings, seeds of *AtWHIRLY1* knockout mutant *why1‐5* contained a lower GSL content compared with the wild‐type seeds (Figure 5b), although the effect of *WHIRLY1* knockout in seeds is milder compared with seedlings. Surprisingly, *why1‐5* seedlings contained reduced amounts of both short‐chain and long‐chain aGSLs compared with wild‐type seedlings, whereas *why1‐5* seeds showed a significantly higher level of short‐chain aGSL but a lower level of long‐chain compounds than wild‐type samples (Figure 5a,b). In other words, knockout of *WHIRLY1* increased the ratio of short‐chain to long‐chain aGSLs in seeds, likely due to an attenuation in chain elongation steps of aGSL biosynthesis during embryo development and seed maturation. Given that all GSLs (except embryo‐synthesized GSLs) are produced in silique and accumulated into embryos continuously as siliques mature by the action of GSL transporters (Nour‐Eldin et al., 2012), the measured GSL content in seed reflects both the GSL biosynthesis rate and the transportation capacity. It remains to be investigated whether WHIRLY1 is involved in regulation of aGSL biosynthesis and seed GSL accumulation in this developmental stage.

It has been reported that the GSL contents in seedlings rise gradually after germination, in which the increase was mainly driven by the biosynthesis of iGSL followed by long‐chain aGSL (Jeschke et al., 2019). In another study, GSL quantity increases slightly 2 days after germination (DAG) under the light growth conditions (in the paper, 4 days on MS medium) followed by a gradual reduction (Meier et al., 2019). In another word, shortly after germination, GSL biosynthesis is activated in young seedlings. The loss‐of‐function of *WHIRLY1* clearly reduces the expression levels of genes involved in aGSL biosynthesis during seedling early development (Figure 4), likely lowering the quantity of newly synthesized aGSL. Moreover, seed‐stored GSLs are hydrolyzed by myrosinases instantly after seed stratification. The protein extract from 24‐h‐stratificated w*hy1‐5* and wild‐type seeds showed high myrosinase activity (Figure 5c). After germination, myrosinase activity in young seedlings declined substantially (Figure 5c), aligning with a previous report that the GSL content in seedlings 2 DAG was higher than in sterile seeds because the biosynthesis rate overpassed the degradation rate (Meier et al., 2019). To date, the preferable GSLs being turned over during seed‐seedling transition and their degrading enzymes are not yet well characterized. Nevertheless, the overall myrosinase activities measured by the rate of sinigrin breakdown are similar between *why1‐5* and wild‐type samples, suggesting that *WHIRLY1* is not involved in regulating GSL hydrolysis by myrosinases. However, measuring myrosinase activity according to sinigrin degradation might not completely reflect the breakdown of GSLs in seedlings. PYK10/BGLU23, a beta‐glucosidase classified as atypical myrosinase, exert the myrosinase activity toward indole GSL (I3G) but not to sinigrin (Ahn et al., 2010). Thus, it cannot be excluded the involvement of other atypical myrosinases in GSL degradation during seedlings development, and that *WHIRLY1* knockout influences these enzyme actions. Indeed, genes encoding atypical myrosinases, including *BGLU19* and *BGLU33*, were significantly induced in the w*hy1‐5* compared with wild‐type seedlings (Table S2), which should be investigated in the future. Furthermore, it is possible that myrosinase‐independent system(s) controlling GLS breakdown exist as there are several studies reporting the fluctuations of GSL contents even without disrupted tissues (Barth & Jander, 2006; Burow, 2016; Martínez‐Ballesta et al., 2013). Nevertheless, it is likely that during the development of *WHIRLY1* knockout seedlings, degradation of seed‐stored short‐chain aGSLs without re‐compensating from *de novo* biosynthesis is one reason leading to lower these metabolite concentrations in *why1‐5* seedlings.

A question is how WHIRLY1 can function in the regulation of the expression of aGSL biosynthesis‐related genes in the early developmental stage. It is known that GSL biosynthesis is regulated precisely at many levels and involves diverse regulators and pathways (Mitreiter & Gigolashvili, 2021). MYB28 is a central transcriptional regulator of aGSL biosynthesis, followed by MYB29 and MYB76 (Sønderby et al., 2010). Interestingly, similar genes were downregulated due to the knockout of *WHIRLY1* as well as in the *MYB28* knockout line (Figure 4a). However, the influence of knockout of *WHIRLY1* on GSL metabolism was only observed at the seedling stage, as there was no difference in the expression levels of GSL‐associated genes in mature *why1‐5* knockout compared with the wild‐type plants (Figure S3). This indicates that during early development, WHIRLY1 acts as an additional regulator of aGSL‐related genes, while in mature plants, aGSL‐related gene expression is regulated without WHIRLY1 and predominantly by MYB28. It has been reported that WHIRLY1 might bind to melted promoter regions of nuclear target genes, whereby it has an affinity to certain binding motifs, such as the ERE including inverted repeat sequences (IR) and W‐boxes (Desveaux et al., 2002, 2005). As expected, the promoters of GSL‐related genes contain several MYB binding site motifs for transcriptional regulation by MYB28 and MYB29, and additionally, many W‐boxes and IR2 sequences that could serve as potential binding sites of WHIRLY1 are distributed throughout promoter sequences (Figure S5). The binding of WHIRLY1 on promoter of these genes could affect their expression level and/or change epigenetic markers in the promoter region.

### WHIRLY evolution and glucosinolate diversification in the order Brassicales

Gene duplication is the primary driving force for evolution as it provides material for natural selection. Duplicated genes could either maintain the ancestral function, become silent (pseudogene), or develop a new function. In the present work, the knockout of *WHIRLY1* strictly affected genes involved in Met‐derived aGSL biosynthesis but did not influence the production of other types of GSLs. Therefore, the co‐occurrence of the novel Met‐derived aGSL biosynthesis and an additional *WHIRLY* locus in the genome (Figure 6; Figure S4) prompts the question of whether the new WHIRLY protein might have obtained a novel function during evolution. Species belonging to core families of the order Brassicales have three WHIRLY proteins instead of two as found in other families of this order (Figure 6). The additional WHIRLY1‐like protein, which is chloroplast‐targeted, might have enabled retrograde control of the first steps of aGSL biosynthesis occurring in chloroplast. Synteny analysis showed that *AtWHIRLY1* and *AtWHIRLY3* share the same ancestral gene with monocot *WHIRLY1*, whereby AtWHIRLY3 seems to be more closely related to the monocot WHIRLY1 proteins than AtWHIRLY1 (Krupinska et al., 2022). Likely, after the duplication in the event At‐β, *AtWHIRLY3* kept the original ancestral gene function, while *AtWHIRLY1* gained a novel function in the regulation of secondary metabolism. Its evolutionary co‐appearance with aGSL biosynthesis‐related genes makes it an ideal candidate for a regulator of chloroplast‐located steps in GSL biosynthesis during seedling development.

## EXPERIMENTAL PROCEDURES

### Plant materials and growth

Seeds of *A. thaliana* Columbia‐0 and mutants were surface‐sterilized in 70% (v/v) ethanol for 5 min and then rinsed several times with sterile water. Seeds were placed on Petri plates containing half‐strength MS media supplemented with 1% (w/v) sucrose and 0.7% (w/v) agar. After 3 days of stratification in darkness at low temperatures, seeds germinated under a continuous light intensity of 15 μE m^−2^ sec at 22 ± 2°C and 60% relative humidity. Seedlings were investigated at 5 days after germination (DAG). To determine morphological characteristics, seedlings were grown vertically and then photographed from the side. A scale was set according to a ruler included in the picture using Fiji ImageJ version 1.53 (Schindelin et al., 2012). Hypocotyl and root were manually marked and their lengths were automatically measured.

### Establishment and genotyping of CRISPR/Cas9‐mediated knockout mutants of AtWHIRLY1

Knockout lines of *AtWHIRLY1* (*At1g14410*) were created by site‐directed mutagenesis using CRISPR/Cas9 (Kumlehn et al., 2018; Li et al., 2021). Annealed oligonucleotides were integrated via *Bbs*I restriction enzyme sites in plasmid pSI57 yielding pGH510. The gRNA/Cas9 expression cassettes were introduced into p6i‐d35S‐TE9 (DNA‐Cloning‐Service, Hamburg, Germany) via *Sfi*I, generating plasmid pGH482. This construct was used for the Agrobacterium‐mediated transformation of the ecotype Col‐0. Primary transformants (T1) were screened on Murashige and Skoog (MS) media supplemented with 33.3 μg mL^−1^ hygromycin B. Resistant seedlings were checked for the presence of DNA encoding the Cas9 endonuclease by PCR with primers Cas9‐F2/Cas9‐R2. The next generations (T2 and T3) of *Cas9*‐positive T1 plants were further analyzed. The mutated *WHIRLY1* locus was screened by PCR amplification and DNA sequencing. The mutated *WHIRLY1* homozygous lines were also checked for the absence of the Cas9 construct using specific primers.

For genotyping of the *WHIRLY1* knockout mutants, a PCR‐coupled cleavage amplified polymorphic sequence (CAPS) approach was used. Firstly, segments containing a mutated site were amplified by PCR, whereby each reaction comprises 1 μL of diluted DNA (100 ng) as the template, 2 μL 10X *Taq* DNA Polymerase Buffer, 0.4 μL 25 mM MgCl_2_, 1.2 μL each primer including Why1_F1 and Why1_R1 (Table S4), and 0.1 μL 10 U μL^−1^ *Taq* DNA Polymerase (EURx, Poland). Then, 5 μL PCR products were digested with restriction enzyme in a 20 μL reaction with 2 μL 10X NEBuffer 3.1 and 1 μL 10 U μL^−1^ *Bsl*I (New England Biolabs, USA). The reaction was performed at 37°C for 3 h. The products obtained after digestion were visualized by electrophoresis on an agarose gel.

### Chlorophyll fluorescence measurements

To measure maximum photosystem II (PSII) efficiency, the FlourCam7 version 1.2.5.3 system (PSI, Czech Republic) was used. Firstly, seedlings were dark‐adapted for 15 min to determine the minimum chlorophyll fluorescence yield (*F*_O_ value). Then, plants were exposed to a saturating flashlight, leading to the maximum fluorescence emission (*F*_M_ value). The maximum PSII value was calculated as the ratio *F*_V_/*F*_M_, where *F*_V_ is the variable fluorescence (*F*_V_ = *F*_M_ − *F*_O_).

### Determination of pigments

To determine pigment contents, 20–30 mg of seedling shoots were collected and finely ground in liquid nitrogen. Then, plant materials were homogenized with 200 μL of acetone/Tris buffer (80% acetone in 10 mM Tris–HCl pH7.8) by vigorously mixing. The mixture was incubated for 10 min in a cold ultrasonic bath before centrifugation at 13 000 rpm for 10 min at 4°C. Next, the supernatant was transferred into a new centrifuge tube. The absorption values of the supernatant were measured using a NanoPhotometer^®^ NP80 (Implen GmbH, Germany) at the following wavelengths: 470, 537, 647, 663, and 750 nm. Pigment concentration was calculated using the equations of Sims and Gamon (2002).

### RNA extraction and quantitative RT‐PCR

For gene expression analysis, 5‐day‐old whole seedlings were ground in liquid nitrogen, and the total RNA was isolated using the RNeasy Plant Mini Kit (QIAGEN, Germany). One microgram of total RNA was used to synthesize cDNA with the RevertAidTM H Minus First Strand cDNA Synthesis Kit (Thermo Fisher Scientific, USA). The quantitative real‐time PCR reaction was conducted using 1 μL cDNA (12.5 ng), 0.4 μL forward/reverse primer, 5 μL 2X SYBR green master mix (KAPA SYBR FAST Universal, KAPABIOSYSTEMS), and the CFX Connect Real‐Time PCR Detection System (Bio‐Rad, USA). Gene expression analysis by qRT‐PCR was performed pairwise between mutant and wild‐type samples of four independent biological replicates, each containing a pool of about 30 seedlings growing on the same plates. The relative expression level was calculated using the method of Pfaffl (2001), by which three reference genes were used: *AtACTIN2*, *At5g46630*, and *AtPP2AA2*. Normalized gene expression level was then normalized to the wild‐type level, which was set as 1. Statistical analysis on gene expression level between mutant and wild type was performed using the normalized values. The gene identifiers (IDs), gene names, gene‐specific primers, and amplicon sizes are provided in Table S5.

### RNA sequencing

Total RNA was isolated using the RNeasy Plant Mini Kit (QIAGEN, Germany). Then, RNA quantity and quality were determined using the Agilent RNA 6000 Pico Kit with Bioanalyzer 2100 system (Agilent Technologies, USA). Samples with high purity and RNA Integrity Number (RIN) were sent to Novogene Co., Ltd (UK) for library preparation, RNA sequencing, and bioinformatics analysis according to their standard protocols. RNA‐seq was done using an Illumina‐based NovaSeq 6000 system with 150 bp paired‐end read sequencing. Reads were checked with FastQC and cleaned up before mapping to the reference *A. thaliana* genome (TAIR10) by HISAT2 (Kim et al., 2019). The number of reads for each gene was counted using featureCounts of HISAT2. Then, the fpkm value (fragments per kilobase of transcript per million mapped reads) was calculated as the relative expression level of a gene in the sample, in which a gene is considered as expressed if the fpkm >1. Differently expressed genes between the mutant and the wild type were determined using raw read counts by DESeq2 R package. Functional annotation of DEGs was performed using the GO enrichment tool (Aleksander et al., 2023; Ashburner et al., 2000).

### Quantification of glucosinolates

Three to five milligrams of *A. thaliana* whole seedlings were ground in liquid nitrogen and subsequently mixed with 450 μL Methanol:Chloroform (2:1 volume ratio) extraction solution supplemented with 20 μg mL^−1^ sinigrin as an internal standard and 200 μL deionized water. Samples were incubated at room temperature for 60 min, followed by centrifugation at 10 000×*g* for 20 min. The upper aqueous phase was used for GSL determination via Liquid Chromatography coupled with tandem Mass Spectrometry (LC–MS/MS). The separation of substrates was performed at 35°C with a Nucleoshell RP18 column (50 × 3 mm, particle size 2.7 μm; Macherey‐Nagel, Germany) using an Agilent 1290 high‐pressure liquid chromatography (HPLC) system. As eluents A and B, water and acetonitrile containing 0.1% formic acid, were used respectively, with 0.5 mL min^−1^ flow rate. Initially, GSLs were separated with 2% of eluent B for the first 0.5 min, which was increased linearly to 95% over the next 7 min. After washing, the column remained at 95% eluent B for 3 min. The starting conditions were restored within the next 0.5 min, and the column was re‐equilibrated with 2% B for 2 min. The analytes were detected by electrospray ionization MS/MS (ESI‐MS/MS) by an API 3200 triple‐quadrupole LC–MS/MS system with an ESI Turbo Ion Spray interface and operated in the negative ion mode (AB Sciex, Germany). The ion source parameters were set as follows: 40 psi curtain gas, −4000 V ion spray voltage, 650°C ion source temperature, 60 psi nebulizing and drying gas. GSL‐specific signals were acquired via Multiple Reaction Monitoring (MRM), with a scan time of 15 msec; Q1 and Q3 masses (Q1, Q3 resolution unit) and compound specific parameters for each analyte are described in Table S6. Peak areas were calculated automatically by IntelliQuant algorithm of the Analyst 1.6 software (AB Sciex, Darmstadt, Germany), with manual adjustment when necessary. GSL content in each sample was calculated in Microsoft Excel, after normalization to the internal standard and to fresh weight. Peak areas were calculated automatically by the IntelliQuant algorithm of the Analyst 1.6 software (AB Sciex, Germany), with manual adjustment when necessary. GSL content in each sample was calculated and normalized to the internal standard and the fresh weight.

### Determination of myrosinase activity by spectrophometric method

To determine the activity of myrosinases, around 100 mg of fresh plant material was frozen in liquid nitrogen and quickly ground into powder. The ground sample was homogenized in 1 mL extraction buffer containing 10 mM of potassium phosphate (pH 6.5), 1 mM EDTA, 3 mM dithiothreitol (DTT), 5% glycerol, and one tablet of cOmplete™, Mini, EDTA‐free Protease‐Inhibitor‐Cocktail (Merck, Germany). After centrifuging at 12 000×*g* for 15 min at 4°C, the supernatant was filtered by passing through syringe filters Chromafil xtra MV‐45/25 0.45 μm (Macherey‐Nagel, Germany) and aqueous solution was collected into a new tube. The extraction was repeated for another two times, and then, filtrates are combined. After the extracted solution was concentrated 10 times using Vivaspin^®^ Turbo 4 PES 10 kDa MWCO (Sartorius, UK), the total protein concentration was determined using the ProtaQuant™ Assay (Serva, Germany).

Myrosinase activity was quantified as the rate of sinigrin degradation by the concentrated protein extract. The reaction buffer contained 33.3 mM potassium phosphate (pH 6.5) and 0.1 mM sinigrin. After equilibrating the reaction buffer at 37°C for 15 min, the reaction started by adding 50 μg of extracted protein into 1 mL of reaction buffer. The decrease in absorbance at 227 nm of the reaction mixture was recorded at 37°C for 30 min. Myrosinase activity was calculated using the formula (*A*_0_ − *A*_t_)/(E*t), where *A*_0_ and *A*_t_ are the initial absorbance and the absorbance after time *t*, respectively; *t* is the reaction time (min); *E* is the molar extinction coefficient, 7500 for sinigrin (Piekarska et al., 2013). The final myrosinase activity is given as nmol of sinigrin degraded by 1 mg of concentrated protein extract per minute.

### Phylogenetic analysis

Putative WHIRLY proteins were identified using BLASTP (Altschul et al., 1990) and HMMER v.3.3.2 (Finn et al., 2011) based on the conserved Whirly domain of AtWHIRLY1 (Pfam: PF08536). First, the BLASTP search with a threshold E‐value of 0.01 was used for the initial identification of the potential WHIRLY protein available in database. Then, the raw HMM profile was downloaded by searching the WHIRLY family in the Pfam database (Mistry et al., 2021), and the WHIRLY proteins were extracted from the protein database by hmmsearch with an E‐value threshold of 1E‐5. Finally, the Pfam was used to verify the presence of a Whirly topology (β‐β‐β‐β‐α‐linker‐β‐β‐β‐β‐α) in their protein structures. Redundant protein sequences, misannotated, and poor‐quality sequences were filtered out. Multiple sequence alignment of putative WHIRLY proteins of all species or Brassicales species was performed by using the ClustalW with default parameters (Thompson et al., 1994). The alignment was manually adjusted and trimmed before using to build a phylogeny tree. The rooted tree was constructed by using MEGA version X (Kumar et al., 2018) with the neighbor‐joining (NJ) method and bootstrap analysis with 1000 replicates. Putative sequences from bacteria and archaea were used as outgroups. The phylogenetic tree was then processed by FigTree v1.4.4 (http://tree.bio.ed.ac.uk/software/figtree/).

### Statistical analysis

Physiological measurements, qRT‐PCR, and GSL quantification were performed with three to four independent biological replicates. The mean of biological replicates was used to calculate the standard deviations and standard errors. A two‐tail paired Student's *t*‐test was performed to estimate the statistically significant differences between lines using Microsoft Excel. Asterisks indicate statistical significance with * for *P*‐value <0.05, ** for *P*‐value <0.01, and *** for *P*‐value <0.001.

## AUTHOR CONTRIBUTIONS

LTN and KH conceived and designed the experiments; SK and GH generated the mutants; AG and KK screened the mutants; LTN performed phenotypic measurement, RNA‐seq analysis, myrosinase assay, phylogenetic analysis, and data analysis; PM, JZ, and SA did the setup, acquisition, and GSL quantification; LTN generated the illustrations; LTN, KH, and KK wrote the manuscript. All authors read and approved the manuscript.

## CONFLICT OF INTEREST

The authors declare no competing interests.

## Supporting information


**Figure S1.** Relative expression level of *AtWHIRLY1* in different T‐DNA insertion mutants by qRT‐PCR. (a) Expression level of *AtWHIRLY1* was investigated by three different primer pairs amplifying three equivalent different segments on the *AtWHIRLY1* mRNA. The value was normalized to *ACT2* level and the transcript level of *AtWHIRLY1* in the wild type was set as 1. (b) Three different mutants, including *why1‐1* (SALK_023713), *why1‐2* (SALK_147680), and *why1‐3* (SALK_03900), have the T‐DNA integrated at the beginning of *AtWHIRLY1* coding sequence. Two ATG codons are highlighted with yellow boxes (Schaller, 2017).
**Figure S2**. Validation of the expression level of aliphatic and indole GSL genes in seedlings by qRT‐PCR. Normalized expression level measured by qRT‐PCR (bar charts on left vertical axis) and normalized read counts by RNA‐seq (line chart on the right vertical axis), both showing the average of mean and error bars showing the standard deviation of three independent biological replicates. Gene expression level in WT was set as 1. Asterisks indicate statistical significant level of Student's *t*‐test between qRT‐PCR‐based normalized expression level of selected genes in the *why1‐5* mutant and wild‐type seedlings, *P‐value <0.05, **P‐value <0.01, ***P‐value <0.001
**Figure S3**. Normalized expression level of aGSL genes in mature plants. The wild‐type and *WHIRLY1* knockout mutant *why1‐5* plants were grown under short‐day condition for 5 weeks. 9th and 11th leaves of mature plants were collected to analyze gene expression level. Gene expression level in WT was set as 1. Bar chart shows the average of mean, and error bars denote standard deviation of four technical replicates, each containing 3 plants.
**Figure S4**. Phylogeny tree of the WHIRLY family. The evolutionary history of WHIRLY family in plants was constructed using the neighbor‐joining method. The rooted bootstrap consensus tree inferred from 1000 replicates, in which the bootstrap values are shown next to the branches. The analysis involved 773 protein sequences, including WHIRLY proteins from various groups, such as vascular plants, ferns, and algae. Protein sequences from bacteria and archaea sharing a similar structure with plant WHIRLIES were treated as outgroup. Selective lineages are highlight in branches and their names are given on the right. Several plant lineages might contain three distinctive WHIRLY proteins, including Brassicaceae, Fabaceae, Salicaceae, Cucurbitaceae, and Aracaceae.
**Figure S5**. Distribution of *cis*‐elements on promoter of GSL‐related genes. The 1000 bp upstream in promoter region of each gene was analyzed by PLACE (https://www.dna.affrc.go.jp/PLACE/?action=newplace). Different motifs are located in both positive strand (up) and negative strand (down). Several MYB binding site motifs were found on the promoter of GSL‐related genes. Additionally, we also identified many W‐boxes and inverted repeat 2 (IR2) sequences, which can serve as potential binding sites for WHIRLY1.

**Table S1.** Summary of RNA‐seq library quality control.
**Table S2**. Differentially expressed genes (DEGs) between the 5‐day‐old *WHIRLY1* knockout mutant and WT seedlings. Significant differentially expressed genes (DEGs) were identified as |log2FC| > 1 and *P*‐value <0.01 and are illustrated in black letters. Genes exhibiting milder changes in their transcript abundances (1 > |log2FC| > 0.5, *P*‐value <0.01) are also presented here in gray letters. Genes involved in different pathways are denoted by different colors, in which green resembles biotic stress response (1), orange, abiotic response (2), blue, developmental process (3), and yellow, RNA metabolism (4). Genes related to sulfur metabolism are highlight yellow box.
**Table S3**. GSL contents in 5‐day‐old *why1‐5* knockout and wild‐type seedlings. The glucosinolate profiles (nmol mg^−1^ FW) of the wild‐type Col‐0 and the knockout *why1‐5* mutant seedlings 5 days after germination, initially grown in 15 μE m^−2^ sec continuously light, 22 ± 2°C. The metabolite concentrations were determined by LC–MS/MS. Descriptive statistics of each biological replicate includes mean, standard deviation (SD), *P*‐value of Student's *t*‐test between *why1‐5* and wild‐type samples.
**Table S4**. GSL contents in the *why1‐5* knockout and wild‐type seeds. The glucosinolate profiles (nmol mg^−1^ FW) in dry seeds determined by LC–MS/MS. Descriptive statistics of each biological replicate includes mean, standard deviation (SD), *P*‐value of Student's *t*‐test between *why1‐5* and wild‐type samples.
**Table S5**. Information of primers used in the study.
**Table S6**. LC/MS–MS detection parameters of glucosinolates.

## Data Availability

All relevant data supporting the results of this article can be found within the manuscript and the supporting materials provided. Raw data from the RNA‐seq samples have been submitted to the National Centre for Biotechnology Information (NCBI) Sequence Read Archive (SRA) database (# PRJNA1101217). The data will be publicly available and accessible at https://www.ncbi.nlm.nih.gov/sra/PRJNA1101217. Please contact the corresponding author for any further information regarding the RNA‐seq data.
