# Supplementary figures and images for: WHIRLY1 regulates aliphatic glucosinolate biosynthesis in early seedling development of Arabidopsis
Source: Plant J. 2024 Dec 3;121(1):e17181. doi: 10.1111/tpj.17181 (PMC11712025; doi:10.1111/tpj.17181)

**Figure S1**

**
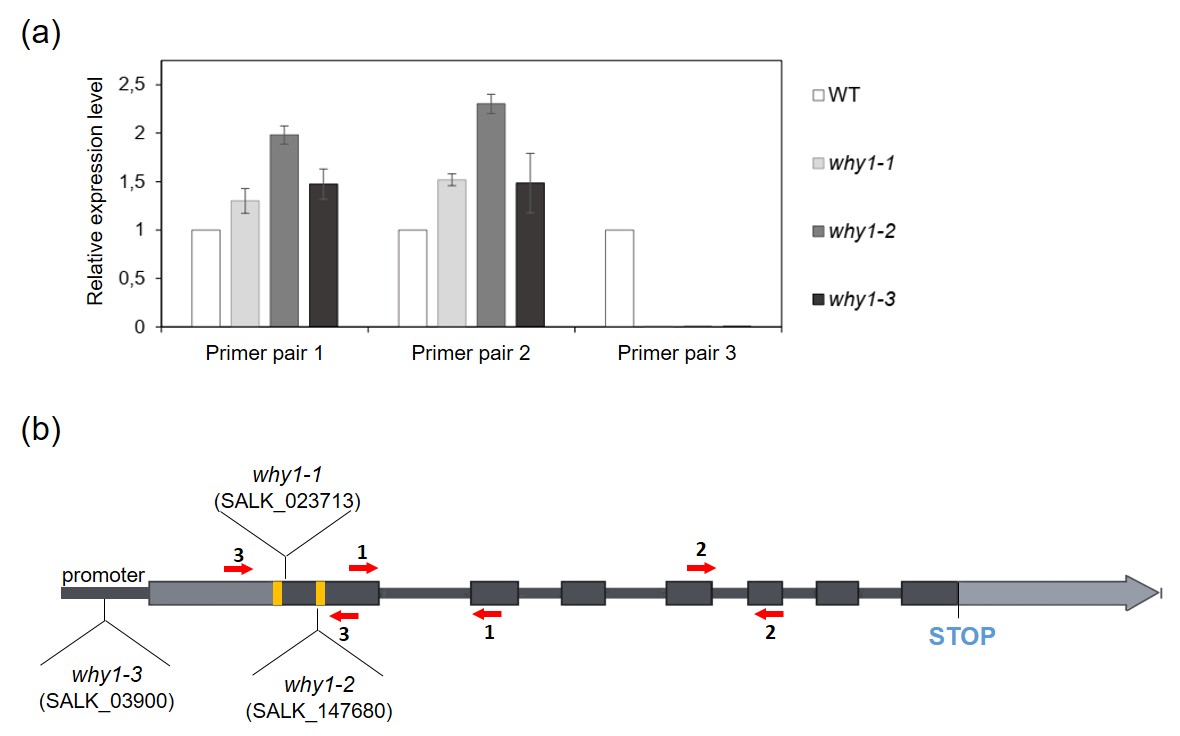
**

**Figure S2**

**
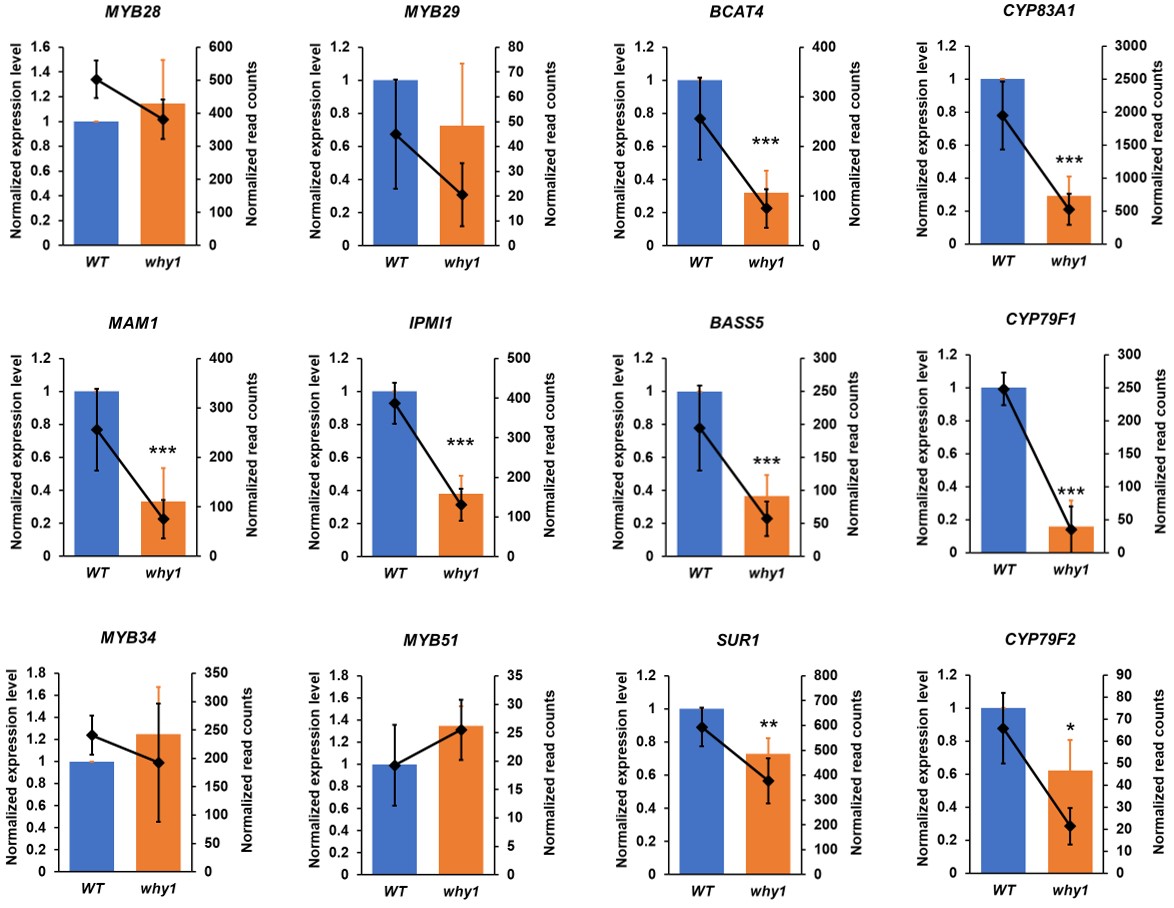
**

**Figure S3**


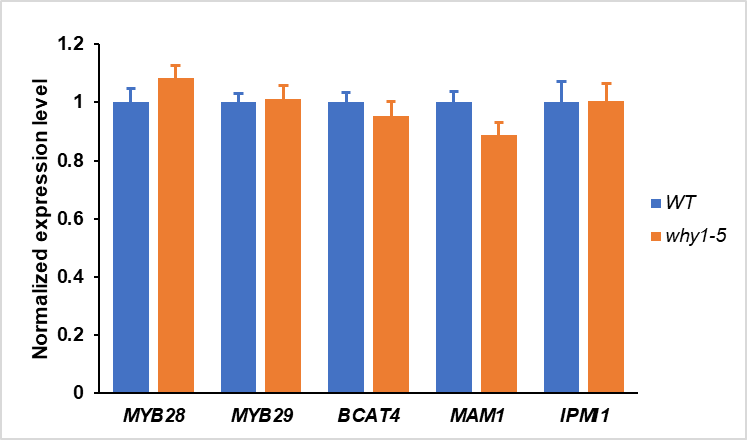


**Figure S4**

**
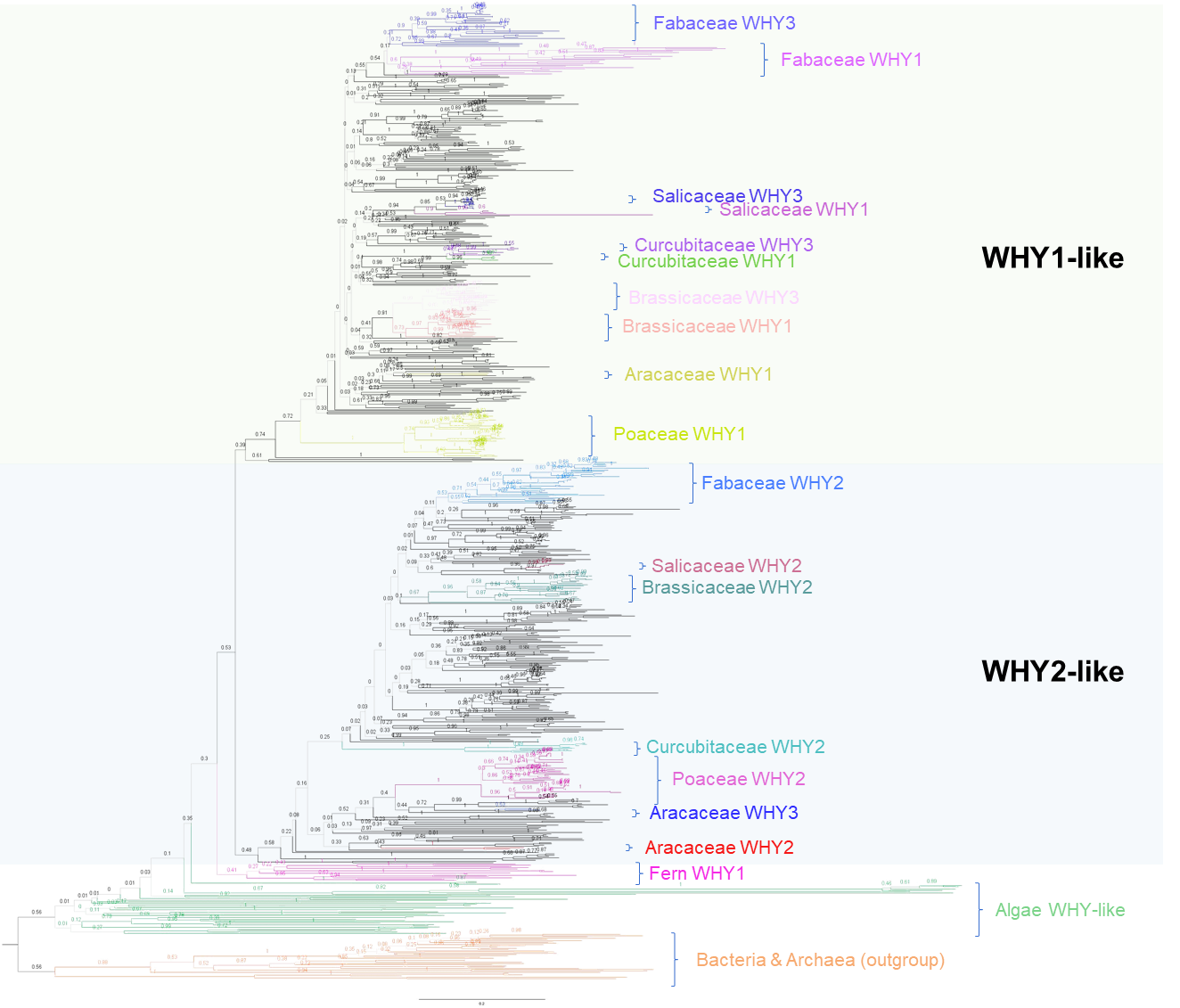
**

**Figure S5**


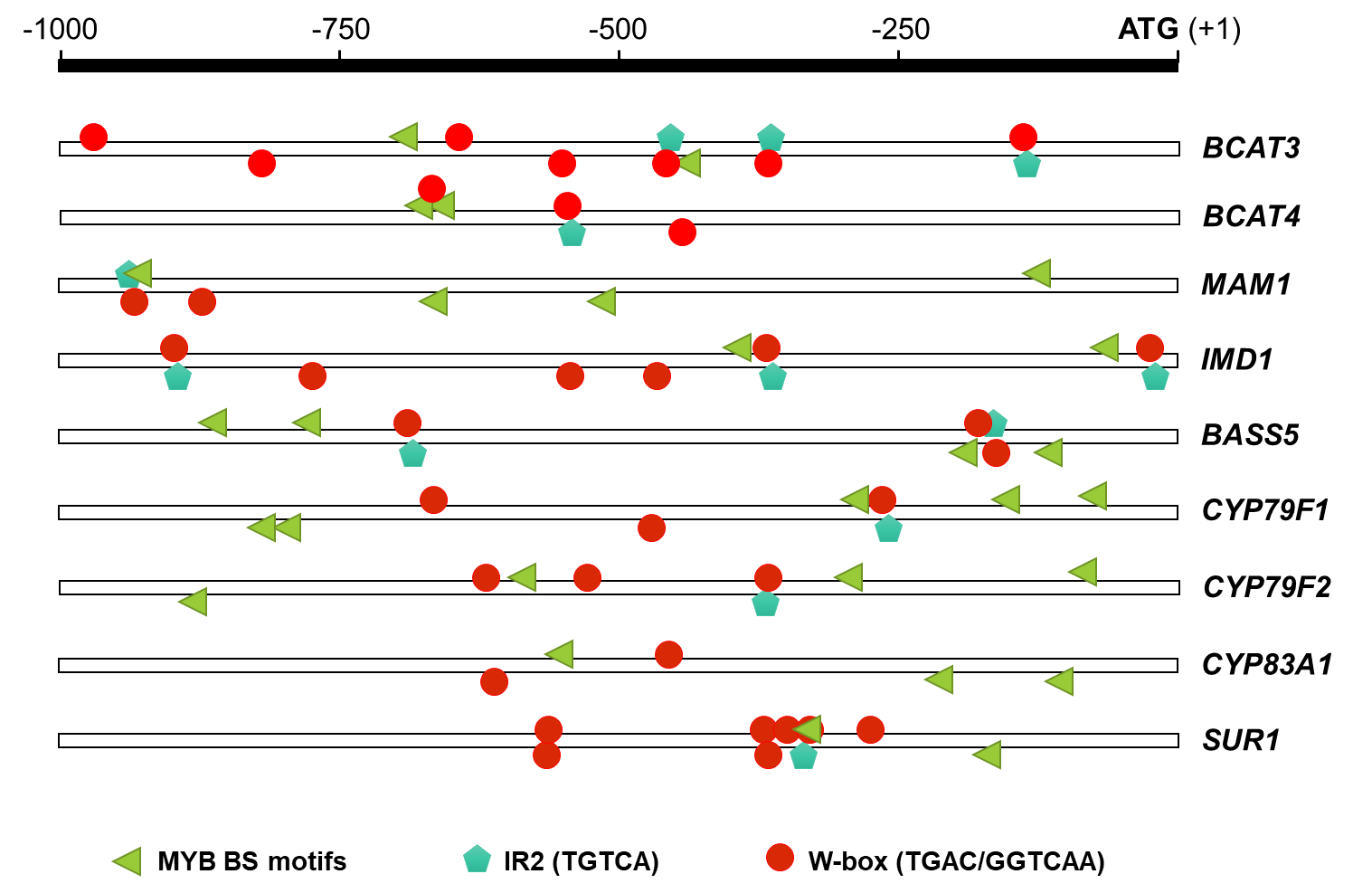

Supplement: Supplementary file 1 — Figure S1. Relative expression level of AtWHIRLY1 in different T‐DNA insertion mutants by qRT‐PCR. (a) Expression level of AtWHIRLY1 was investigated by three different primer pairs amplifying three equivalent different segments on the AtWHIRLY1 mRNA. The value was normalized to ACT2 level and the transcript level of AtWHIRLY1 in the wild type was set as 1. (b) Three different mutants, including why1‐1 (SALK_023713), why1‐2 (SALK_147680), and why1‐3 (SALK_03900), have the T‐DNA integrated at the beginning of AtWHIRLY1 coding sequence. Two ATG codons are highlighted with yellow boxes (Schaller, 2017). Figure S2. Validation of the expression level of aliphatic and indole GSL genes in seedlings by qRT‐PCR. Normalized expression level measured by qRT‐PCR (bar charts on left vertical axis) and normalized read counts by RNA‐seq (line chart on the right vertical axis), both showing the average of mean and error bars showing the standard deviation of three independent biological replicates. Gene expression level in WT was set as 1. Asterisks indicate statistical significant level of Student's t‐test between qRT‐PCR‐based normalized expression level of selected genes in the why1‐5 mutant and wild‐type seedlings, *P‐value <0.05, **P‐value <0.01, ***P‐value <0.001 Figure S3. Normalized expression level of aGSL genes in mature plants. The wild‐type and WHIRLY1 knockout mutant why1‐5 plants were grown under short‐day condition for 5 weeks. 9th and 11th leaves of mature plants were collected to analyze gene expression level. Gene expression level in WT was set as 1. Bar chart shows the average of mean, and error bars denote standard deviation of four technical replicates, each containing 3 plants. Figure S4. Phylogeny tree of the WHIRLY family. The evolutionary history of WHIRLY family in plants was constructed using the neighbor‐joining method. The rooted bootstrap consensus tree inferred from 1000 replicates, in which the bootstrap values are shown next to the branches. The ana [file TPJ-121-0-s001.docx]
